# Ge11-Modified pH-Sensitive Polymer Micelles: A New Breakthrough in Targeted Therapy for Non-Small-Cell Lung Cancer

**DOI:** 10.3390/pharmaceutics18040498

**Published:** 2026-04-17

**Authors:** Xingmeng Ma, Zhu Wang, Jingyi Wang, Xingyu Chen, Jinggang Zhang, Dengxue Yang, Shiyi Xu, Xueying Yan

**Affiliations:** School of Pharmacy, Heilongjiang University of Chinese Medicine, Harbin 150040, China

**Keywords:** polypeptide GE11, doxorubicin, pH-responsive micelle, EGFR, lung cancer

## Abstract

**Background/Objective**: In lung cancer treatment, increasing the concentration of antitumor drugs at the tumor site, enhancing efficacy, and reducing systemic toxicity are significant challenges. This study aims to develop an intelligent responsive polymer micelle system (GPDD) that achieves efficient accumulation and controlled release of drugs at lung tumor sites through targeted and pH-responsive design. **Methods**: The GPDD system is formed by the self-assembly of GE11-PEG-hyd-DOX conjugates and co-loads free DOX. This system utilizes the targeting effect of the GE11 peptide with the epidermal growth factor receptor (EGFR) to accumulate at the tumor site, while the hydrazone bond serves as a pH-responsive linker that breaks in the acidic tumor microenvironment, triggering drug release. Experiments employed CCK-8 cytotoxicity assays and tumor-bearing nude mouse models (strain not specified) for in vitro and in vivo evaluations. **Results**: In vitro experiments showed that GE11-modified GPDD effectively inhibited tumor cell growth. In tumor-bearing nude mouse experiments, GPDD demonstrated more significant tumor suppression effects and lower systemic toxicity compared to free DOX and unmodified PDD. **Conclusions**: The GPDD nanocarrier integrates targeting and pH responsiveness, improving antitumor efficacy and reducing side effects, with translational potential. The novelty of the study lies in its dual-functional design and co-loading strategy, providing new insights for tumor-targeted delivery systems.

## 1. Introduction

Lung cancer is a type of cancer that begins to form when abnormal cells grow in the lungs in an uncontrolled manner [1], which can cause serious injury or even death and seriously threaten human health. Non-small-cell lung cancer (NSCLC) and small-cell lung cancer (SCLC) are the two main clinicopathologic types of lung cancer, of which NSCLC is more common, accounting for 80–85% of cases [2]. DOX is a cyclic nonspecific drug with a wide antitumor spectrum and has killing effects on a variety of tumors in various growth cycles. It is used in the treatment of breast cancer, ovarian cancer, non-small-cell lung cancer, etc. [3,4,5]. However, DOX can produce a wide range of biochemical effects on the body and has a strong cytotoxic effect [6,7,8]. Due to its poor selectivity, DOX also has the same killing effect on cells in normal tissues [9,10,11]. In severe cases, DOX will cause myocardial damage and cardiotoxicity leading to heart failure, so its clinical application is limited [12,13,14]. Therefore, it is crucial to develop drug delivery strategies that can improve efficacy and reduce toxic side effects.

In recent years, with the continuous in-depth exploration of tumor treatment, molecular targeting technology has developed rapidly. The research of targeted drug delivery systems (TDDSs) has become one of the key and difficult points in pharmaceutical research in the world. TDDSs, also known as targeted preparations, are a new preparation technology and process method. The mechanism of TDDSs in the treatment of cancer means that drugs can selectively reach specific physiological parts, organs, tissues or cells through systemic circulation, and play a therapeutic role in this target site [15,16]. Compared with other kinds of drug therapy, TDDSs can enhance drug targeting ability [17], reduce the toxic and side effects of drugs on the body [18], control drug release, improve the stability of drug action [19], change the half-life of drugs [20], reduce drug dosage and frequency, and improve the comprehensive effectiveness of clinical drugs [21,22]. Because of their many advantages, they are favored by the clinic and have epoch-making significance.

Tumor cells or tumor blood vessels overexpress certain receptors, such as epidermal growth factor receptor (EGFR), Transferrin receptor (TfR), Folate receptors (FRs), etc. [23,24,25]. These receptors are not expressed or are expressed in small amounts in normal cells [26]. EGFR is overexpressed on the surface of NSCLC and is an important target for the treatment of NSCLC [27,28,29]. At present, EGFR-targeted drugs such as gefitinib, afatinib and ocittinib are the first-line treatment drugs recommended in many targeted therapy guidelines [30,31,32]. Although these drugs have specific targeting properties and better therapeutic effects, many NSCLC patients will develop acquired drug resistance within about 1 year of taking them [33,34,35]. Therefore, it is very important to seek new EGFR-targeting drugs for the targeted therapy of NSCLC. With the gradual deepening of research on targeted drugs and the further expansion of the range of molecular targets [36,37,38], Cheng et al. confirmed that the corresponding ligand of EGFR can assist cell internalization through the mesh protein-mediated endocytosis pathway to enable drugs to enter tumor cells and finally achieve EGFR target-mediated active targeting [39,40].

GE11 is a small-molecule polypeptide obtained by phage peptide library screening technology, which is composed of 11 amino acids (YHWYGYTPQNVI) [41]. Studies have found that GE11 has a high affinity for tumor cells with a high expression of EGFR, and can be specifically combined with it [42,43,44]. Compared with natural macromolecular ligands, GE11 has the advantages of low molecular weight, low immunogenicity, easy diffusion and high targeting [45]. The application of GE11 to targeted drugs for cancer diagnosis and treatment is increasing and the effect is outstanding, which has broad application prospects in the future [46,47].

**Figure 1:**
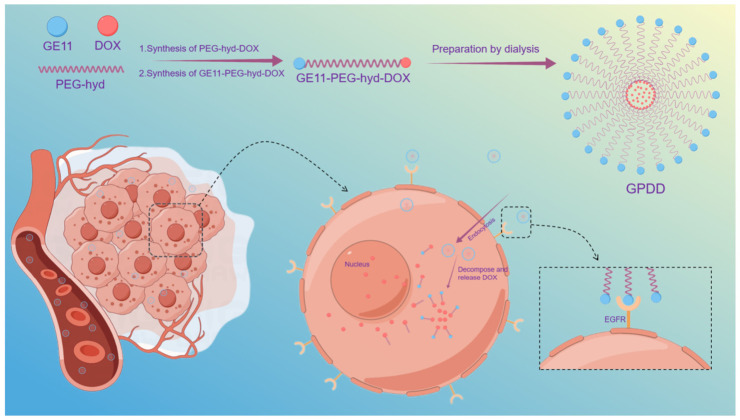
Diagram of GE11-PEG-hyd-DOX/DOX (GPDD) micellar drug release.

Based on the above research background and technological trends, this study aims to construct and evaluate a novel pH-sensitive active targeting polymeric micelle system (GPDD). This system uses PEG-PLA micelles as the core platform, connects DOX to the end of the PEG chain via a hydrazone bond (pH-sensitive arm), and modifies the micelle surface with the GE11 peptide as the targeting head group, while also co-encapsulating free DOX. This design integrates the following multiple functions: (1) utilizing the specific binding of GE11 to the epidermal growth factor receptor (EGFR) to achieve active targeting; (2) leveraging the weakly acidic microenvironment of tumor tissues to trigger hydrazone bond cleavage, enabling precise controlled drug release; (3) enhancing overall drug accumulation at the tumor site through the classic micelle system’s EPR effect and long-circulation characteristics. This study systematically validates the targeting ability, pH-responsive drug release properties, and enhanced efficacy with reduced toxicity of GPDD against NSCLC through in vitro and in vivo experiments, aiming to provide a promising nanotechnology solution to overcome the limitations of traditional chemotherapy and existing targeted therapies. GPDD reaches the tumor site through a specific interaction between GE11 and EGFR (Figure 1). In the tumor microenvironment, pH-sensitive hydrazone bonds are broken, GE11-PEG-hyd and doxorubicin are separated, micelles are divided, and the inclusion of doxorubicin is released to enhance the therapeutic effect.

## 2. Materials and Methods

### 2.1. Materials and Reagents

Aladdin Industrial Corporation (Shanghai, China) provided Doxorubicin hydrochloride (HCPT, purity > 98%), alkaline phosphatase inhibitor, chloral hydrate, hematoxylin dye, Ponceau dye, RIPA lysate, alkaline phosphatase inhibitors, protease inhibitor, SDS-PAGE protein loading, PBS buffer (5×), and sealing solution (10×). Hangzhou Gutuo Biotechnology Co., Ltd. (Hangzhou, China) supplied Polypeptide GE11 (CysYHWYGYTPQNVI). Hangzhou Xinqiao Biotechnology Co., Ltd. (Hangzhou, China) supplied Mal-PEG-NH2. Shanghai Maclin Biochemical Technology Co., Ltd. (Shanghai, China) supplied 4-nitrophenyl chloroformate and 4-dimethylaminopyridine. Wuhan Sanying Biotechnology Co., Ltd. (Wuhan, China) supplied BAX Monoclonal antibody (60267-1-lg), GAPDH Monoclonal antibody (60004-1-lg), Bcl2 Monoclonal antibody (8103-1-lg), and p62/SQSTM1 Monoclonal antibody (66184-1-lg). Wuhan Aibotek Biotechnology Co., Ltd. (Wuhan, China) supplied HRP-conjugated Goat anti-Rabbit IgG. Roce Biotechnology Co., Ltd. (Suzhou, China) supplied the Cell Death Detection Kit POD. Biyuntian Biotechnology Co., Ltd. (Shanghai, China) supplied the BCA protein detection kit. Harbin Shiguo Biological Technology Co., Ltd. (Harbin, China) supplied FBS, DMEM/F-12 medium, and penicillin–streptomycin dual antibiotic solution. Sigma Corporation, USA (Ronkonkoma, NY, USA), supplied the dialysis bag (MW: 2000/3500) and Methanol (chromatographic grade). All other reagents were of analytical grade, and all experiments were conducted using distilled water.

Beijing Weitong Lihua Laboratory Animal Co., Ltd. (Beijing, China) supplied BALB/c-nude mice. All animal experiments were carried out following the Heilongjiang University of Chinese Medicine Guidelines for Animal Experimentation, and the institution’s Animal Ethics Committee approved the protocol.

### 2.2. Preparation and Characterization of GPDD

#### 2.2.1. Synthesis of Graft PEG-NPCF

Stoichiometric amounts of DMAP, NPCF, and Maleimide-polyethylene glycol-amino (Mal-PEG-NH2, 2000 Da) with a molar ratio of 3:1.2:1 were, respectively, weighed and placed in designated vessels with a specific volume of DMSO solution until the three components were completely dissolved, after which the mixture was stirred magnetically at room temperature for 24 h. Upon completion of the reaction, the solution was transferred into a pre-treated dialysis membrane (MWCO: 2.0 kDa), and the product was freeze-dried after 24 h of dialysis against deionized water to obtain the graft product PEG-NPCF.

#### 2.2.2. Synthesis of Graft PEG-hyd

An appropriate volume of DMSO was added to the flask containing PEG-NPCF and hydrazine hydrate, which were weighed at a ratio of 2:3. After initial dissolution, the mixture was heated to 65 °C in a water bath under reflux conditions with continuous stirring for 24 h, after which the reaction mixture was transferred to a dialysis bag (MWCO: 2.0 kDa). The grafted product, PEG-hyd, was obtained by freeze-drying following 24 h of dialysis against deionized water.

#### 2.2.3. Synthesis of Graft PEG-hyd-DOX (PD)

A certain amount of DOX·HCl was weighed and placed in a drying container, DMSO was added to dissolve the solution, and a triethylamine solution (two equivalents relative to DOX·HCl) was added dropwise to the mixture. The suspension was magnetically stirred for 24 h at room temperature, then transferred to a centrifuge tube and centrifuged for 10 min (3000 r/min); this operation was repeated. Once the supernatant was completely removed, the resulting precipitate was lyophilized to obtain deacidified DOX powder. PEG-hyd (mass ratio 2:3) and deacidified DOX were weighed and placed in a round-bottom flask, DMSO was added to dissolve them completely, an appropriate amount of triethylamine was added, and the mixture was refluxed in a water bath at 65 °C with continuous stirring for 24 h. Finally, the reaction mixture was transferred into a dialysis bag (MWCO: 2.0 kDa). After 24 h of pure water dialysis, the unreacted DOX was removed with 0.8 μm filtration membrane, and the filtrate was lyophilized to obtain the graft product PEG-hyd-DOX (PD).

#### 2.2.4. Synthesis of Graft GE11-PEG-hyd-DOX

**Figure 2:**
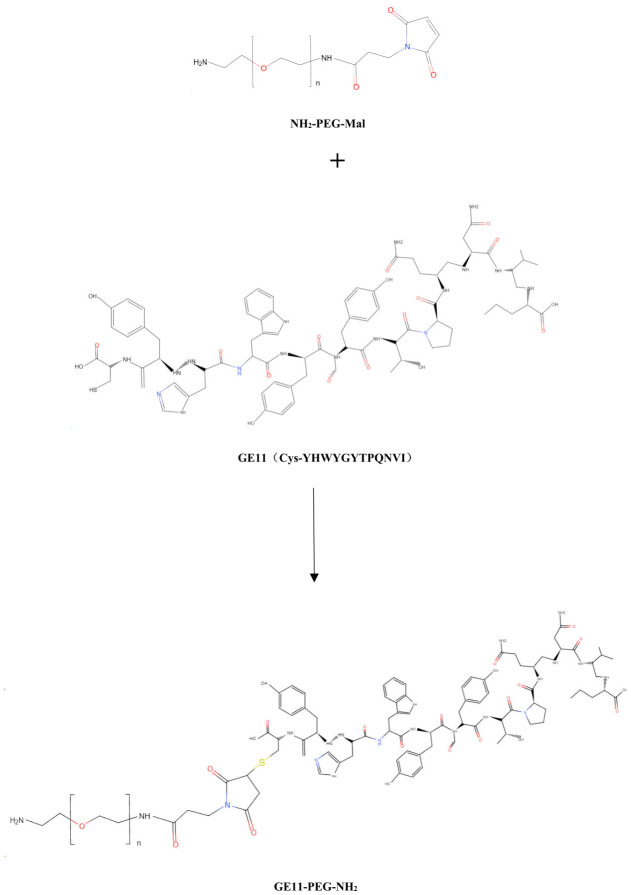
Synthetic scheme of GE11-PEG-NH_2_.

Mal-PEG-NH_2_ and polypeptide GE11 (Cys-YHWYGYTPQNVI) with a mass ratio of 2:3 [48] were weighed to prepare synthetic grafts based on the addition reaction between Maleimide and sulfhydryl groups. The grafts were magnetically mixed at room temperature for 24 h. The reaction products were transferred to a dialysis bag (MW: 2.0 kDa) and dialysis was performed in pure water for 24 h. The reaction products were collected and lyze-dried to obtain the graft product GE11-PEG-NH_2_ (Figure 2).

Then, according to the method under the preparation process “Section 2.2.1, Section 2.2.2 and Section 2.2.3,” the conjugate containing GE11, GE11-PEG-hyd-DOX (GPD) was synthesized.

#### 2.2.5. GPDD Micelles Were Prepared by the Dialysis Method

An appropriate amount of deacidified DOX was weighed and dissolved in anhydrous DMSO to prepare a 1.00 mg/mL DOX solution in anhydrous DMSO for further use. A graft micelle solution (2.50 mg/mL) was prepared by dissolving lyophilized graft GPD powder in deionized water. The micelles were fully mixed and emulsified by ultrasonication for 5 min (2 s on/3 s off cycle). Using a DOX-to-GPD mass ratio of 1:20, the aforementioned deacidified DOX was slowly added to the graft micelle solution, and the mixture was stirred magnetically (500 rpm) for 2 h. Subsequently, the solution was placed in a dialysis bag (MWCO: 3.5 kDa) and dialyzed against deionized water for 24 h under dark conditions [49]. Aqueous dialysis was used instead of organic solvents to dissolve block copolymers and drugs, and the solution in the dialysis bag was centrifuged at low temperature for 10 min (3000 r/min). Uncoated DOX precipitated at the bottom of the centrifuge tube, and the supernatant was collected and diluted to a final volume of 5 mL with deionized water to obtain the drug-carrying GPDD micelles [50].

#### 2.2.6. Particle Size, Zeta Potential, and Morphology of Micelle

The hydrodynamic diameter, polydispersity index (PDI), and zeta potential of GPDD formulations were characterized using a Zetasizer (Nano-ZS90; Malvern Instruments, Malvern, UK). To visualize morphological features, samples were negatively stained with 2% sodium phosphotungstate prior to imaging by transmission electron microscopy (TEM) on an HT7700 system (Hitachi, Tokyo, Japan).

#### 2.2.7. Encapsulation Efficiency and Drug Loading

Based on the cleavage of hydrazone bonds in an acidic environment (pH = 6.0), a small amount of hydrochloric acid was added to the prepared drug-carrying micelles GE11-PEG-hyd-DOX/DOX (GPDD) solution, and the pH was adjusted to 6.0 to break the hydrazone bonds, thereby releasing DOX from both the grafted segments and the micellar core. The encapsulation rate (EE) and drug loading capacity (DL) were measured. The GPD graft solution was obtained, and the grafting rate of DOX (G) was calculated based on the weight ratio of DOX to the GPD graft after acid-induced cleavage under the aforementioned conditions. The encapsulation efficiency and drug loading capacity of DOX in the GPDD drug-loaded micelle solution, as well as the grafting rate of the GPD graft solution, were calculated, and these measurements were performed in triplicate. The formulas for calculating the encapsulation efficiency, drug loading capacity, and grafting rate of the drug-loaded micelle graft solution are as follows:


$$\mathrm{EE}\;(\%)=W_{\mathrm{drug}}/W_{\mathrm{cast}}$$

$$\mathrm{DL}\;(\%)=W_{\mathrm{drug}}/W_{\mathrm{total}}$$

$$G\;(\%)=W_{\mathrm{Marry}\mathrm{drug}}/W_{\mathrm{Marry}}$$

In the equation, EE—encapsulation efficiency; DL—drug loading; G—graft ratio; W_drug_—weight of conjugated and encapsulated drug; W—total drug weight; W_total_—GPDD total weight; W_marry drug_—GPD drug weight; and W_marry_—total weight of GPD.

#### 2.2.8. In Vitro Drug Release

DOX and GPDD were prepared into 1 mg/mL solutions with PBS, and 1 mL of each of the above two solutions was accurately pipetted and placed in 49 mL of release medium with pH values of 5.0, 6.8, and 7.4 (each containing 5% SDS), respectively, and shaken in a constant-temperature water bath shaker (37 °C, 100 rpm). At preset times of 0.25 h, 0.5 h, 1 h, 2 h, 4 h, 8 h, 12 h, 24 h, 48 h and 72 h, 1 mL of solution in the dialysis medium was taken, and an equal volume of fresh, pre-warmed medium was added to determine the content of DOX and calculate the cumulative release rate (Q) by the formula:


$$Q(\%)=(V_{0}\cdot C_{t}+V\cdot \sum_{n-1}^{t-1}C_{t})\cdot 100\%\cdot W^{-1}$$

C_t_: Drug concentration in the release medium measured at each time point (mg/mL).

W: Total weight of drug input (mg).

V_0_: Total volume of releasing media (mL).

V: Volume per sample (mL).

### 2.3. Cell Culture

This study used A549 cells. Xuan Yan Biotechnology Co. (Shanghai, China) supplied the A549 cells. At 37 °C with 5% CO_2_, A549 cells were cultured in F-12 media with 10% FBS. Before experiments, cells were precultured to 80% confluence.

### 2.4. Western Blot

The expressions of P62, Bax and Bcl-2 in the control group, free DOX, PEG-hyd-DOX/DOX (PDD) and GPDD were detected by immunoimprinting. A549 cells were collected, RIPA lysate was added, ice lysated for 30 min, centrifuged for 30 min (4 °C, 1000 rpm), and the supernatant was absorbed to obtain the total protein extract of A549 cells. The proteins extracted from each group were equally loaded onto SDS-PAGE gel for electrophoresis. After electrophoresis, they were transferred to PVDF membrane. After transfer, the PVDF membrane was cleaned with TBST, incubated with sealing solution for 1 h at room temperature, cleaned with TBST, primary antibody was added, and the membrane was incubated at 4 °C overnight. The dilution ratio of primary antibody was GAPDH (1:50,000), BAX (1:5000), Bcl-2 (1:2000), p62 (1:2000). After cleaning with TBST, the second antibody was added and incubated for 2 h. The dilution ratio of the second antibody was: IgG-HRP goat anti-rabbit (1:2000). Regarding exposure, the ECL luminescent liquid was prepared 1:1 and mixed in the light shielding dish. The film was placed in the light shielding dish so that the front of the film was fully in contact with the luminescent liquid. The chemiluminescence imaging system was used for exposure and the protein bands after exposure were preserved. For image analysis, the band strength was quantified using ImageJ software (version 2022). ImageJ software was used to quantify the intensity of protein bands in each group, and GAPDH was used as the internal reference to calculate the ratio of gray values between the target protein and the corresponding internal reference bands, so as to reflect the relative content of target protein.

### 2.5. Cytotoxicity Assay

To evaluate the cytotoxic profiles of free DOX, PEG-hyd-Dox/DOX (PDD), and GPDD, we employed the CCK-8 assay. Initially, cells were seeded into 96-well plates at a density of 1 × 10^4^ cells per well and allowed to adhere for 12 h. Subsequently, the culture medium was replaced with 100 μL of fresh media supplemented with graded concentrations of DOX (1, 5, 10, 20, or 50 µg/mL) for a 48 h exposure period; untreated wells within the same plate functioned as negative controls. Following treatment, 10 µL of CCK-8 solution (5 mg/mL in PBS) was introduced to each well, and plates were incubated for an additional 2 h under standard conditions (37 °C, 5% CO_2_). Finally, absorbance at 450 nm (OD_450_) was quantified using a BioTek H1 microplate reader (Refudi Biomedical (Shanghai) Co., Ltd., Shanghai, China).

The cell viability ratio was estimated using the equation below:


$$\mathrm{cell}\;\mathrm{proliferation}\;\mathrm{inhibition}\;\mathrm{rate}(\%)=(1-\frac{{\mathrm{OD}}_{450(\mathrm{control}\;\mathrm{group})}-{\mathrm{OD}}_{450(\mathrm{Drug}\;\mathrm{group})}}{{\mathrm{OD}}_{450(\mathrm{control}\;\mathrm{group})}-{\mathrm{OD}}_{450(\mathrm{Blank}\;\mathrm{group})}})\times 100\%$$

### 2.6. Cellular Uptake Assay

Fluorescence inverted microscopy was employed to monitor the temporal uptake kinetics of GPDD in A549 cells. Briefly, A549 cells were seeded into 24-well plates at a density of 2.5 × 10^4^ cells/mL and incubated for 24 h to ensure adherence. Subsequently, the supernatant was aspirated and replaced with fresh DMEM/F-12 medium supplemented with GPDD, whereas control wells received an equivalent volume of complete culture medium alone; each experimental condition was performed in triplicate. Following additional incubation periods of 4, 8, and 12 h, the media were discarded, and the monolayers underwent three successive rinses with phosphate-buffered saline (PBS). Nuclear counterstaining was then achieved by treating the cells with 4′,6-diamidino-2-phenylindole (DAPI; 5 µg/mL) for 5 min. After a final PBS wash, cellular fluorescence was immediately visualized and documented using a fluorescent inverted microscope.

### 2.7. Apoptosis Assay

Adhering strictly to the manufacturer’s guidelines, we utilized the Annexin V-FITC/PI apoptosis detection kit (Beyotime, Shanghai, China). A549 cells were seeded into six-well plates at a density of 1.5 × 10^6^ cells per well. Upon reaching the logarithmic growth phase, the cultures received treatment with either GPDD or DOX injection (prepared identically to the CCK-8 assay) at a final concentration of 1 μg/mL. Following a 48 h incubation period, cells were harvested via trypsinization (EDTA-free) and washed three times with phosphate-buffered saline (PBS). Subsequently, the cell pellet was resuspended in 300 μL of 1× binding buffer and stained with 5 μL of Annexin V-FITC alongside 5 μL of propidium iodide (100 μg/mL), ensuring all procedures were conducted under light-protected conditions. After a 15 min incubation, an additional 200 μL of fresh 1× binding buffer was introduced to each sample prior to analysis by flow cytometry.

### 2.8. In Vivo Tumor Targeting Evaluation

#### 2.8.1. Tumor Implantation

The establishment of the BALB/c nude mouse model followed protocols from prior investigations. A549 cells were suspended in F-12 medium at a concentration of 5 × 10^7^ cells/mL. Subsequently, 0.2 mL of this cellular suspension was injected subcutaneously into BALB/c-nude mice to induce tumor formation. Experimental interventions commenced once tumor volumes reached approximately 100 mm^3^. All procedures regarding animal husbandry and experimental manipulation adhered strictly to the guidelines established by the Committee on the Use and Management of Experimental Animals at Heilongjiang University of Chinese Medicine.

#### 2.8.2. In Vivo Imaging

Fluorescence imaging was conducted using an IVIS^®^ Lumina XR Series III system (PerkinElmer, Hopkinton, MA, USA) with excitation and emission wavelengths set at 495 nm and 520 nm, respectively. Images were captured at multiple time points post-administration, specifically at 10 min, 30 min, 1 h, 2 h, 4 h, and 8 h. Following the in vivo timeline, ex vivo fluorescence imaging of resected tumors and major organs—including the heart, liver, kidneys, lungs, and spleen—was performed under identical instrumental settings. Quantitative analysis of fluorescence intensity within both tumor tissues and organs was subsequently carried out using the Living Image software (4.4) associated with the IVIS^®^ Lumina XR platform.

#### 2.8.3. Antitumor Efficacy

Tumor-bearing mice were randomly allocated into four cohorts, each comprising five animals. The control cohort received phosphate-buffered saline (PBS), whereas the remaining three groups underwent tail vein administration of doxorubicin (DOX), PDD, or GPDD at a dosage of 10 mg/kg on days 0, 3, 6, 9, 12, 15, and 18. Body mass and tumor dimensions were assessed triennially; tumor volume was calculated employing the equation: V = (L × W^2^)/2. On day 14, mice were euthanized, and tumors alongside livers, kidneys, lungs, spleens, and hearts were excised. Following tumor weighing, specimens were fixed in 4% neutral buffered formalin for 48 h. Subsequently, standard hematoxylin and eosin (H&E) staining procedures were applied to evaluate histological architecture across all organs. Lastly, TUNEL assays were performed on tumor sections to quantify apoptotic indices among the distinct therapeutic regimens.

#### 2.8.4. Drug Distribution In Vivo

Tumor-bearing nude mice were randomly divided into three groups, (1) DOX, (2) PDD, and (3) GPDD, with five samples in each group (*n* = 5). A dose of DOX of 10 mg·kg^−1^ was administered through the tail vein. Samples were collected from nude mice at 6 time points after 10 min, 0.5 h, 1 h, 2 h, 4 h and 8 h. The drug concentration in tissues at each time point was calculated by HPLC analysis.

### 2.9. Statistical Analysis

Group comparisons were conducted using Student’s *t*-test, with data presented as mean ± standard deviation (SD). Results yielding *p*-values below the designated significance thresholds (* *p* < 0.05, ** *p* < 0.01) were deemed statistically significant.

## 3. Results and Discussion

### 3.1. Characterization of GPDD

#### 3.1.1. Morphology and Particle Size Distribution of GPDD

**Figure 3:**
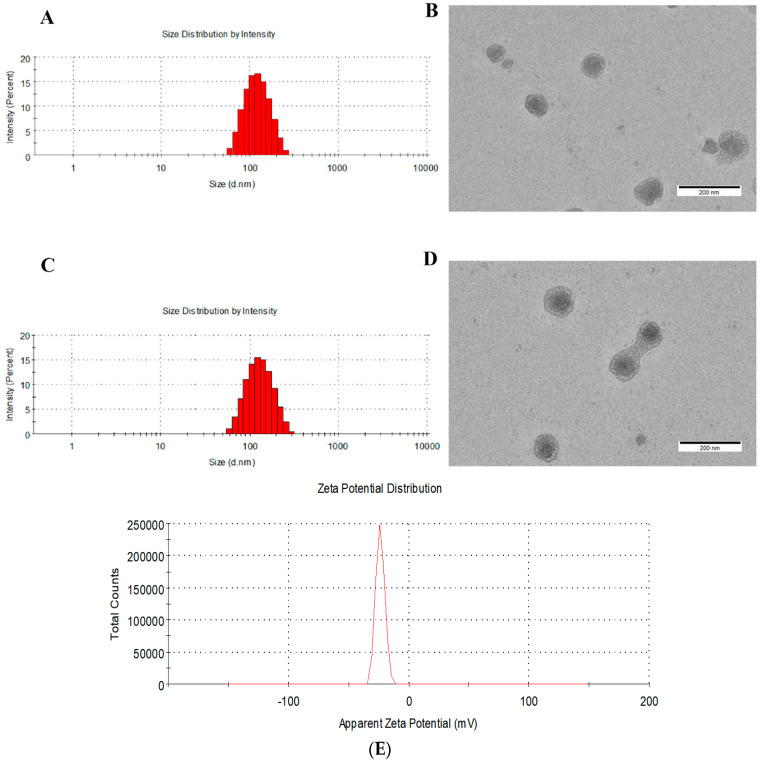
The morphology and size distribution of micelle. (**A**,**C**) GPDD particle size map, (**B**,**D**) GPDD morphology map, (**E**) potential map.

Figure 3A,C illustrate the morphological characteristics and size distribution of GPDD, revealing a uniform spherical structure and excellent dispersibility. The average particle size is approximately 120 nanometers, with a polydispersity index (PDI) below 0.157. Notably, compared to unmodified micelles, GE11-functionalized micelles exhibit a slight increase in hydrodynamic diameter. The anionic nature of the GE11 peptide imparts a negative zeta potential to the nanoparticles, a key attribute that helps mitigate lung sequestration and prevents rapid clearance by cells expressing scavenger receptors. Figure 3E shows the potential diagram of GPDD, with a Zeta potential of −23.5 mV. The encapsulation efficiency of doxorubicin (DOX) remains stable at around 80%, confirming effective drug loading within the micelle core. Surface charge is a critical determinant of nanoparticle behavior; specifically, a slightly negative zeta potential is essential for avoiding lung retention associated with cationic surfaces and evading immediate uptake by the immune system. Furthermore, particle size is another key parameter influencing efficacy. While the optimal cutoff for systemic circulation is generally considered to be below 200 nanometers, the threshold for extravasation into the tumor interstitium has been established at approximately 400 nanometers. Therefore, the physicochemical properties of the synthesized nanoparticles indicate that they are well-suited for targeted tumor drug delivery.

#### 3.1.2. Hydrogen Nuclear Magnetic Resonance (1H-NMR) Analysis of GE11-PEG-NH2

**Figure 4:**
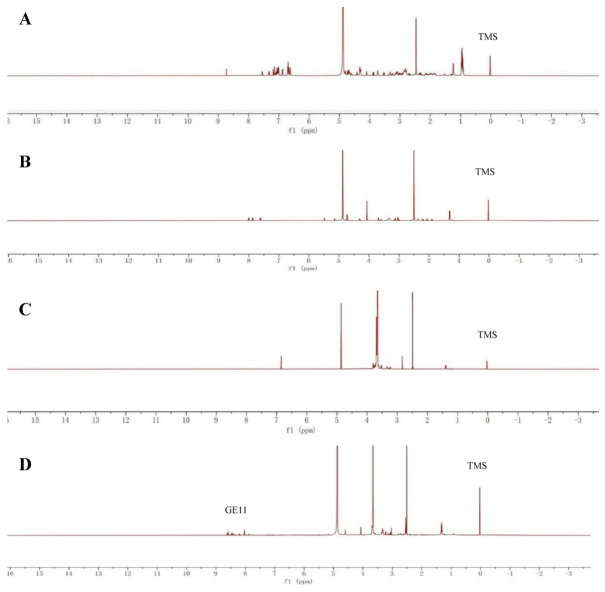

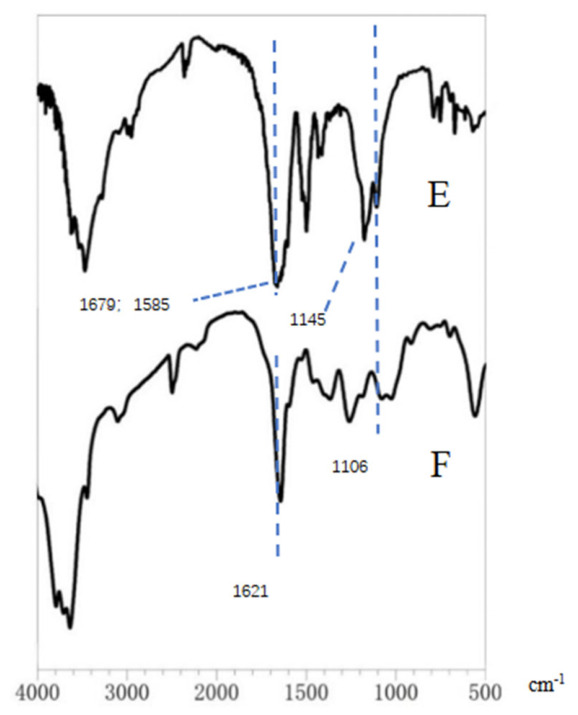
^1^H-NMR of GE11 (**A**), DOX (**B**) and Mal-PEG-NH_2_ (**C**) and GPD (**D**). FTIR spectrum of GPD (**E**) and PD (**F**).

Figure 4A—Characteristic signals of the GE11 peptide: Complex multiplets appearing in the chemical shift range of δ 6.64–8.73 ppm are attributed to the aromatic protons (e.g., benzene ring protons of tyrosine Tyr and histidine His) and amide protons (-CONH-) of the amino acid residues in the GE11 peptide chain. These signals constitute the “fingerprint region” of the GE11 peptide, and their final appearance in the product spectrum is key evidence of successful conjugation.

Figure 4B—Characteristic signals of Doxorubicin (DOX): Signals at chemical shifts δ 3.33 ppm and δ 4.07 ppm are attributed to characteristic protons of the sugar ring moiety in the DOX molecule (e.g., -O-CH_3_) and methylene/methine protons near the anthraquinone ring, respectively. These are markers for identifying the presence of the DOX unit.

Figure 4C—Characteristic signals of the functionalized PEG intermediate (PEG-Mal): δ 3.66 ppm (singlet, integration ~180H): This is the strong characteristic singlet of protons in the repeating unit -O-CH_2_CH_2_- of the polyethylene glycol (PEG) chain, demonstrating the presence of the long PEG chain. δ 6.85 ppm (singlet, integration 2H): This is the characteristic signal of the two equivalent protons on the double bond of the maleimide end group (Mal). This group is the key active site for the subsequent Michael addition reaction with the thiol group (-SH) on GE11.

Figure 4D—Key changes in reaction signals (decisive evidence), with disappearance of the characteristic peak: Compared to Figure 4C, the characteristic singlet at δ 6.85 ppm attributed to the double bond protons of maleimide (Mal) in Figure 4D has completely disappeared. This directly and strongly proves that the maleimide group at the PEG terminus has fully participated in the reaction, i.e., it has undergone a Michael-thiol addition reaction with the thiol group (-SH of -Cys) on the GE11 peptide.

Appearance and shift of characteristic peaks: Regarding the appearance of GE11 signals, complex signals reappear in the aromatic and amide proton region (δ 6.64–8.73 ppm) of Figure 4D, which matches the signal characteristics of GE11 in Figure 4A, confirming that the GE11 peptide has been successfully introduced into the molecular structure. Shift of DOX signals: The characteristic proton peaks of DOX in Figure 4D shift to δ 2.68 ppm and δ 3.66 ppm (compared to 3.33 and 4.07 ppm in Figure 4B). This significant change in chemical shift is due to alterations in the surrounding electron cloud density and chemical environment after DOX is linked to the PEG chain via a hydrazone bond (-hyd-), providing indirect evidence that DOX has been successfully conjugated via a chemical bond rather than simple physical mixing. Retention of PEG backbone signal: The strong singlet of the PEG backbone at δ 3.66 ppm remains present, indicating that the skeletal structure of PEG remains intact during the reaction.

#### 3.1.3. FTIR Analysis of GE11-PEG-NH2

The analysis and determination of two substances at different wavelengths were carried out by Fourier Transform infrared spectroscopy. Figure 4E is the graft product GPD, while Figure 4F is the graft product PD. The absorption peak of the hydrazone bond is 1621 cm^−1^, and both substances have absorption here, indicating that a hydrazone bond exists here. In addition, 1145 cm^−1^ and 1106 cm^−1^ are the absorption peaks of C-O-C stretching vibration (δ_as_) in matrix PEG and the overlapping peaks of hydroxyl stretching vibration (ν_OH_) in polypeptide GE11. Furthermore, 1679 cm^−1^ and 1585 cm^−1^ are the absorption peaks of (ν_C=O_) and (δ_NH_ + ν_CN_) in the amide bond, respectively. The synthesis of FTIR absorption peaks further indicates that the grafting GPD-containing hydrazone bond is successfully synthesized.

#### 3.1.4. Determination of the Coupling Rate Between GE11 and Mal-PEG-NH2

Due to the modification of cysteine groups in the N-terminal of the polypeptide, disulfide bonds will be formed between cysteine molecules in GE11 during the reaction between the polypeptide GE11 and Mal-PEG-NH2, which affects the sampling analysis and coupling rate determination. Therefore, it is necessary to introduce the reducing agent dithithreitol (DTT) into the experiment to break the disulfide bond between protein molecules to open the GE11 dimer. Before HPLC sampling analysis, the suitable proportion of reducing agent DTT was added to the graft solution, and the mixture was fully shaken to make it evenly mixed. After the appropriate reaction time, the GE11 dimer could be completely removed to determine the GE11 and Mal–PEG–NH2 coupling rate of 88.00%. (Table 1).

#### 3.1.5. Determination of GPDD Encapsulation Rate, Drug Loading and Grafting Rate of Grafts

Encapsulation rate (EE), drug load (DL) and grafting rate (G) were determined by HPLC (Table 2). The results show that the GPDD sample has a high encapsulation rate and good stability, which meets the requirements. The grafting rate of DOX in graft GPD is 18.35%.

#### 3.1.6. Stability Study of GPDD

The in vitro stability of micelles constitutes a pivotal prerequisite for their successful deployment in both laboratory-based and living-system biomedical applications. Furthermore, it is imperative that these nanocarriers maintain structural integrity while circulating within the bloodstream. Experimental observations indicated that the micellar formulations exhibited excellent dispersibility following one month of storage at 4 °C; notably, both their mean hydrodynamic diameter and polydispersity index (PDI) remained remarkably consistent throughout this period (Table 3). Moreover, assessments of serum stability in vitro confirmed that these constructs possess sufficient robustness to withstand the conditions encountered during systemic circulation.

#### 3.1.7. Hemolysis Test

**Figure 5:**
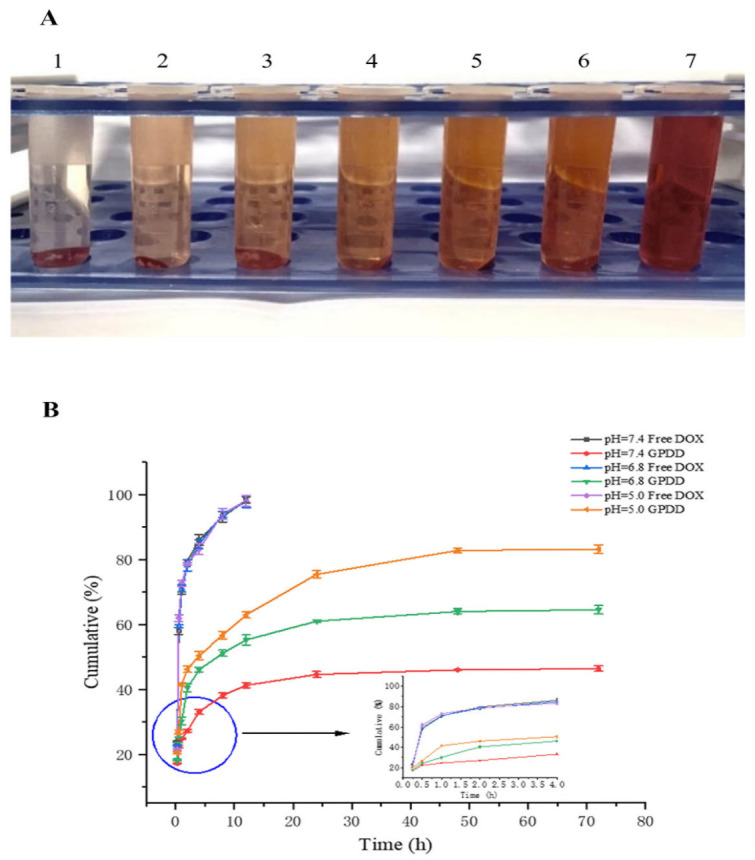
(**A**) Picture of hemolytic experiment. (**B**) In vitro release profiles of DOX and GPDD.

To assess the biocompatibility of the nanocomposite, a hemolysis assay was conducted (Figure 5A; Table 4). Given that the hemolytic percentages for samples in centrifuge tubes 2 through 6 remained below the 5% threshold, the synthesized GPDD demonstrated excellent biosafety.

#### 3.1.8. In Vitro Release of Micelles

The release of free DOX and drug-carrying GPDD micelles in PBS solution is shown in Figure 5B. The release of free DOX in the free group was rapid from 0 to 8 h, and was basically complete at 12 h, reaching about 98%. The release of free DOX did not change with the change in pH value. The drug release of drug-carrying GPDD micelles showed a trend of being rapid first and then slow release, which was greatly affected by pH. The results showed that under the acidic pH = 5.0 environment, the cumulative release of the drug after 24 h could reach 75.46%, and the final release rate reached 83.15%. In a slightly acidic environment with pH = 6.8, the cumulative release of the drug after 24 h reached 61.02%, and the final release rate reached 64.47%. In a neutral environment with pH = 7.4, the cumulative release of the drug after 24 h reached 44.62%, and the final release rate reached 46.34%. The reason for the different release rates between these environments is the cleavage of hydrazone bonds in the acidic environment, which leads to a greater release of DOX. The above data fully confirm that micellar preparations have a good sustained release effect, and the maximum release amount is achieved at pH = 5.0, which makes it reasonable to speculate that they have more powerful antitumor effects. Different mathematical models (zero-order kinetic equation, first-order kinetic equation, Higuchi equation, Weibull equation, and Ritger–Peppas equation) were used to fit the cumulative release percentages of free DOX and GPDD at each time point. The results of the fitting equations and the results of the goodness of fit (R^2^) are shown in Table 5, Table 6 and Table 7. According to the curve of each data model obtained from the fitting results, the fitting condition and release characteristics were analyzed, and the release mechanism reflected by the equation was discussed. Under the conditions of pH = 7.4, pH = 6.8 and pH = 5.0, the release rules of both free drug and GPDD micelle formulations were in accordance with the Weibull equation, and the fitting correlation coefficients were between 0.9787 and 0.9951.

### 3.2. Cell Uptake Assay

**Figure 6:**
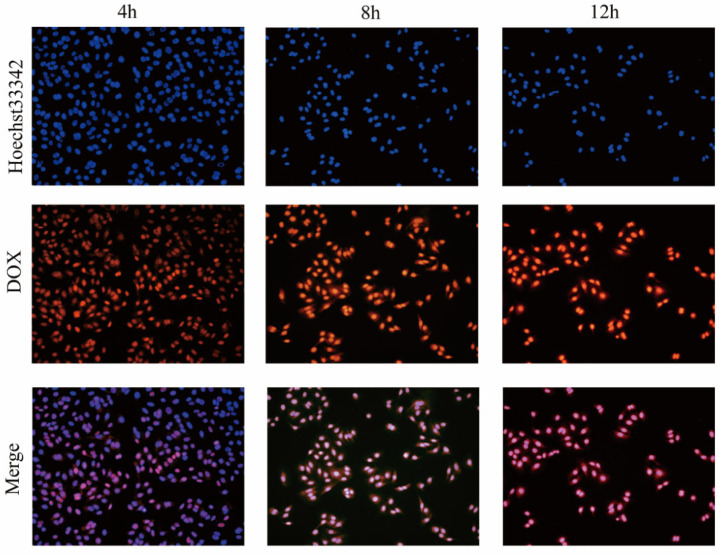
Uptake of GE11-PEG-hyd-DOX/DOX-loaded micelles by A549 cells after incubation for 4, 8, and 12 h (blue is the Hoechst 33342 labeled nucleus, red is the fluorescence of DOX).

Hoechst 33342 is a blue fluorescent dye that penetrates cell membranes and is less toxic to cells, binding to double-stranded DNA in the nucleus to act as a marker. Hoechst 33342 combined with double-stranded DNA has a maximum excitation wavelength of 350 nm and a maximum emission wavelength of 461 nm. DOX supported in GPDD drug-carrying micelles has self-fluorescence, which can be used to investigate the uptake of GPDD drug-carrying micelles by A549 cells using inverted fluorescence microscopy. The results, as shown in Figure 6, demonstrate that DOX release behavior in A549 cells was obviously good after 12 h of co-incubation, which was significantly higher than the results of 4 h and 8 h of co-incubation, indicating efficient cellular uptake of DOX. The degree of uptake was positively correlated with the incubation time.

### 3.3. Western Blot Assay

**Figure 7:**
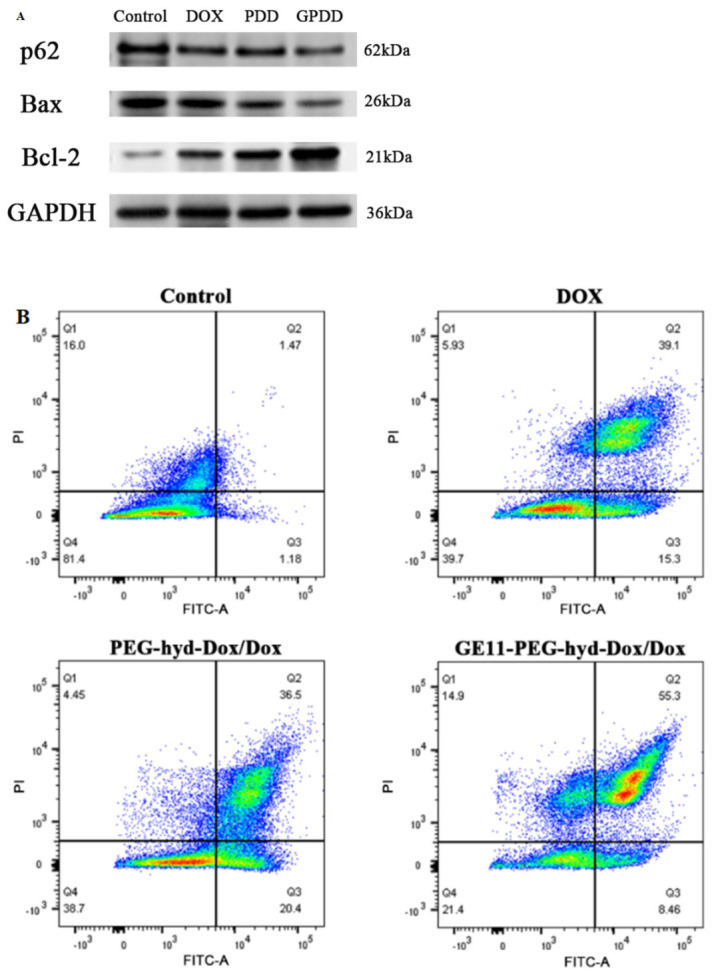

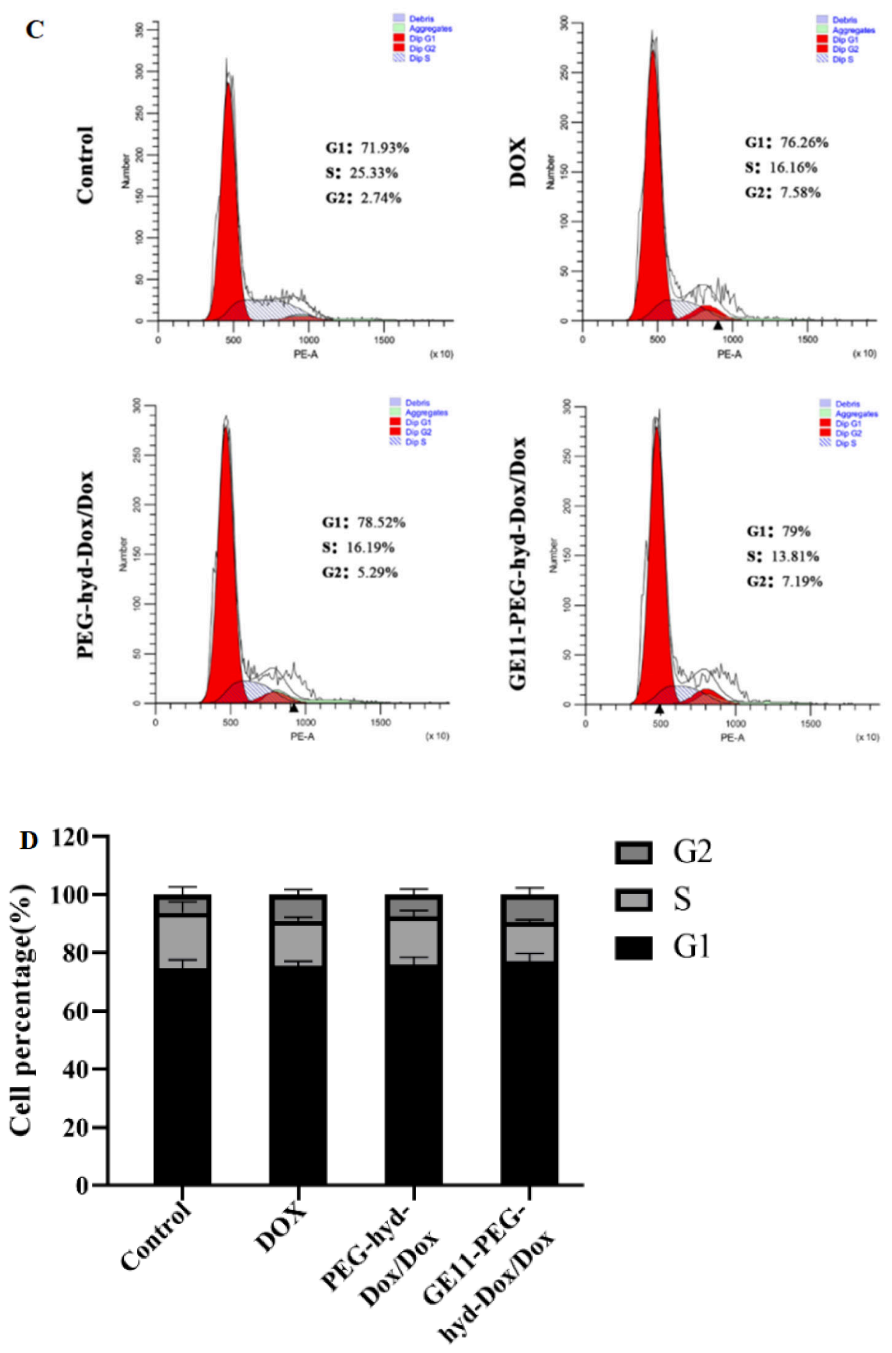
(**A**) Western blot analysis results of p62, Bax, and Bcl-2 proteins in A549 cells. (**B**) Flow cytometry analysis for apoptosis of A549 induced by DOX (3.7 μg/mL), PEG-hyd-DOX/DOX (DOX 3.7 μg/mL) or GE11-PEG-hyd-DOX/DOX (DOX 3.7 μg/mL), respectively. (**C**) Flow cytometry detection of the effects of free DOX, PDD, and GPDD-loaded micelles on the cell cycle of A549 cells after 48 h of action. (**D**) Statistical chart of cell cycle distribution under different groups.

The results of Western blot analysis are shown in Figure 7A and Table 8. Compared with the control group, the expression of p62 protein in the free DOX group, PDD group and GPDD group gradually decreased (*p* < 0.01). The expression of Bcl-2 protein decreased gradually (*p* < 0.01, *p* < 0.05), while the expression of Bax protein increased gradually (*p* < 0.01). In the PDD group, compared with the free DOX group, P62 protein expression was decreased, Bcl-2 protein expression was significantly decreased (*p* < 0.05), and Bax protein expression was significantly increased (*p* < 0.05). Compared with the free DOX group, the expression levels of p62 and Bcl-2 protein in the GPDD group were significantly decreased (*p* < 0.01), and the expression levels of Bax protein were significantly increased (*p* < 0.01). The results showed that GPDD could downregulate the expression of anti-apoptosis protein Bcl-2 and autophagy protein p62. Upregulated expression of the pro-apoptotic protein Bax was observed. The differences in protein expression among the groups support the results of subsequent experiments.

### 3.4. Apoptosis Assay

The effect of GPDD on the apoptosis of A549 cells was detected by flow cytometry (Figure 7B). The lower-right quadrant showed early apoptotic cells with Annexin V-FITC-positive staining and PI-negative staining. In the upper-right quadrant, there are necrotic or late apoptotic cells, i.e., Annexin V-FITC-positive cells and PI-positive cells. Flow cytometry showed that GPDD micelles had the strongest apoptotic effect on A549, and only 21.4% of the cells survived.

### 3.5. Cell Cycle Assay

Flow cytometry was used to investigate the relationship between GPDD-induced autophagy of A549 cells and pericellular duration. In this experiment design, A549 cells were divided into the control group, DOX group, PDD group and GPDD group, and the cell cycle distribution was detected by a PI single staining method. The experimental results are shown in Figure 7C,D. In the control group, the ratio of the G1 stage was 74.76 ± 2.58%, S stage was 19.22 ± 3.16%, and G2 stage was 6.026 ± 2.39%. In the GPDD group, the proportion of the G1 stage was 77.10 ± 2.45%, G2 stage was 9.388 ± 2.06%, and S stage was 13.51 ± 0.69%. There was no significant difference in cell cycle in the DOX, PDD and GPDD groups compared with control group.

### 3.6. Antitumor Effect In Vitro

**Figure 8:**
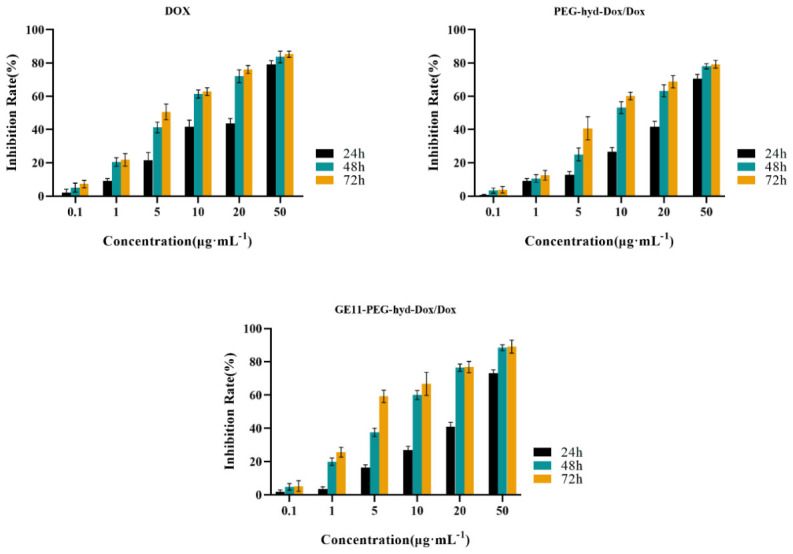
Inhibition of different experimental groups on A549 cells (*n* = 6).

The inhibitory effects of DOX, PDD and GPDD drug-carrying micelles at different concentrations on the proliferation of A549 cells are shown in Figure 8, and the IC50 values of A549 cells in the three experimental groups are shown in Table 9.

The toxicity of free DOX, PDD and GPDD on A549 cells was detected by CCK-8 assay. According to the proliferation inhibition rate data of A549 cells in different experimental groups, it can be seen that within the experimental concentration range, free DOX, PDD and GPDD had obvious cell inhibition effects on A549 cells, and they showed a concentration dependence. When the concentration of free DOX and DOX in drug-carrying micelles was 50 μg/mL, the inhibition rates of A549 cells were 85.33%, 79.32% and 89.12%, respectively. Compared with free DOX drugs and PDD drug-carrying micelles, GPDD drug-carrying micelles showed stronger cytotoxicity to A549. According to the IC50 calculation results, the cell inhibition rate of the GPDD drug-loaded micelle group was higher than that of the free DOX group and PDD drug-loaded micelle group at 24 h, 48 h and 72 h after 72 h administration. The IC50 values of the three experimental groups were 4.89 μg/mL, 7.63 μg/mL and 3.71 μg/mL, which were all higher than the IC50 values at 24 h and 48 h, respectively. It was proved that the inhibitory effect of free DOX, PDD drug-carrying micelle and GPDD drug-carrying micelle on A549 cells was dose-dependent and accompanied by time extension.

### 3.7. In Vivo Targeting Evaluation

**Figure 9:**
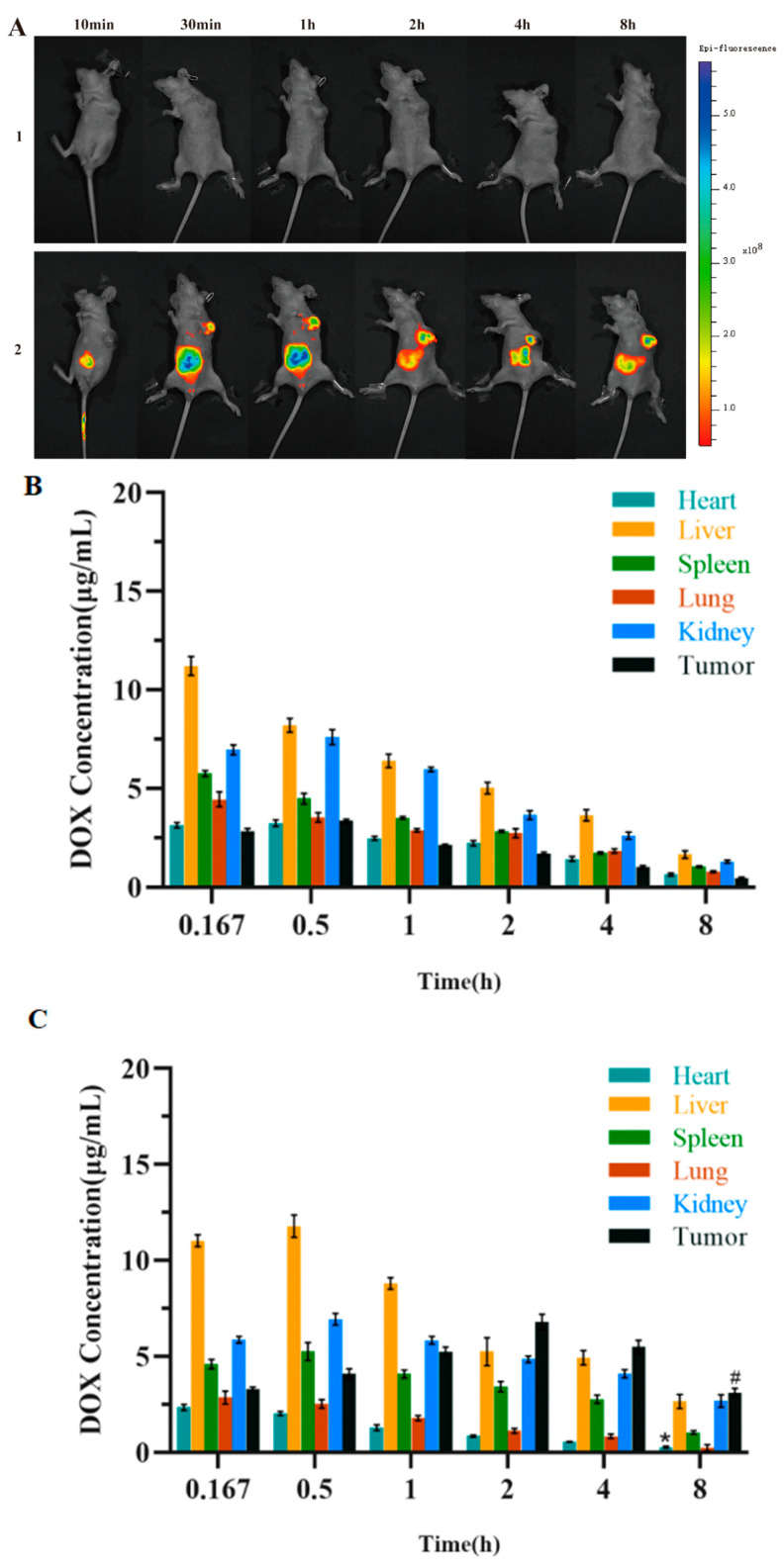

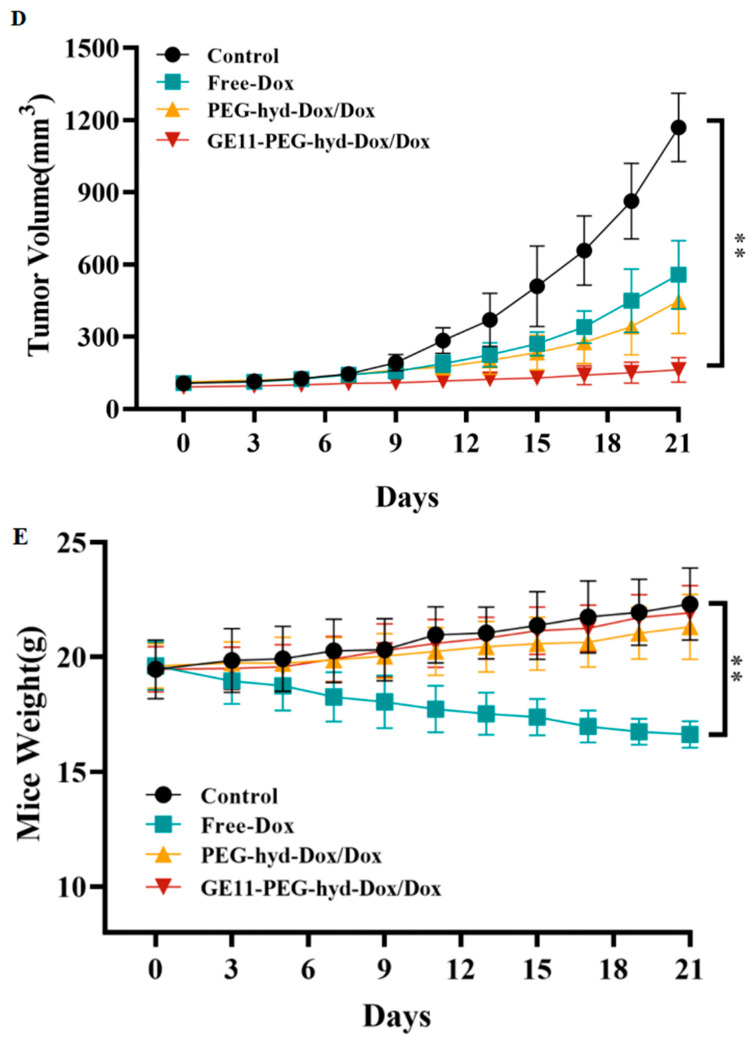

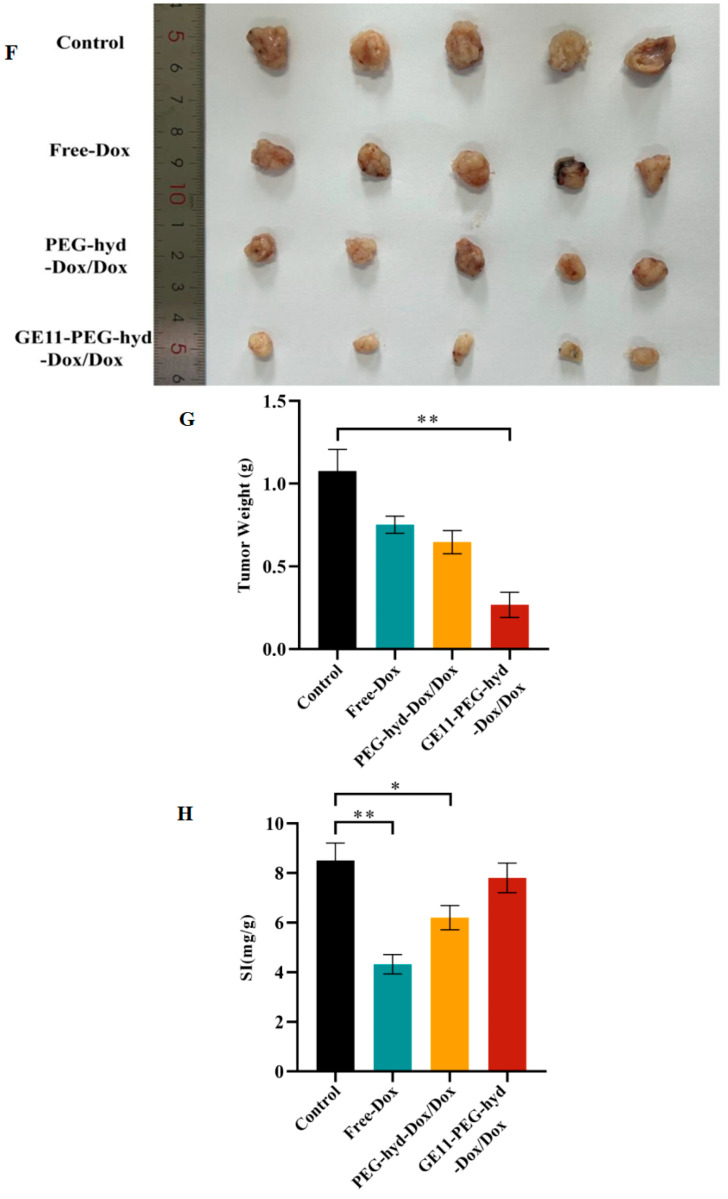

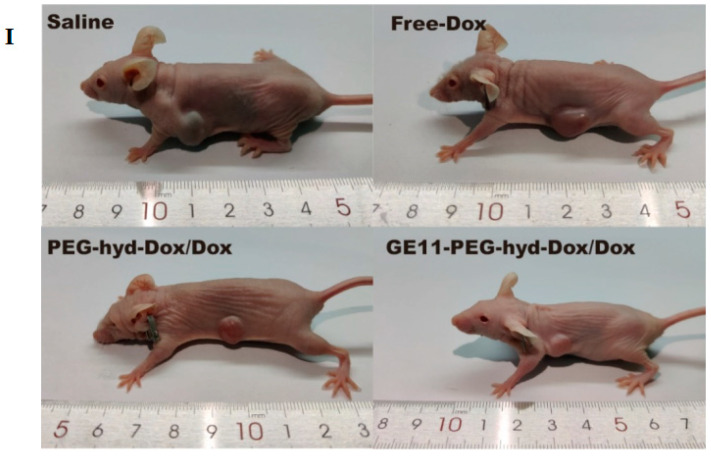
(**A**) In vivo imaging of different experimental groups in tumor-bearing nude mice (*n* = 5): (**1**) control group and (**2**) FITC-labeled GPDD group. (**B**) Visual diagram of DOX in vivo distribution experiment results. (**C**) Visual diagram of the experimental results of GPDD in vivo distribution. (**D**) Tumor growth curve of different groups (*n* = 5). (**E**) Body weight of nude mice after administration (*n* = 5). (**F**) Tumor images of nude mice after treatment. (**G**) Tumor weights of different groups (*n* = 5). (**H**) Spleen index of different groups (*n* = 5). (**I**) Representative photographs of different experimental groups in tumor-bearing nude mice. ((**C**),* *p* < 0.05 compared with the drug distribution in the heart of the free DOX administration group at 8 h, # *p* < 0.01 vs. drug distribution in the tumor at 8 h in the free DOX group compared with the drug distribution in the tumor of the free DOX administration group at 8 h) ((**D**), ** *p* < 0.01, control compared with GPDD) ((**E**),** *p* < 0.01, free DOX, GPDD separately compared with control) ((**G**),** *p* < 0.01, control compared with GPDD) ((**H**), * *p* < 0.05, ** *p* < 0.01, free DOX, PDD,GPDD separately compared with control).

In this study, in vivo targeted fluorescence images of nude mice with A549 non-small-cell lung cancer in different drug administration groups were collected through the small animal live imaging system (Figure 9(A1,A2)) to study the biological distribution of FITC-labeled GPDD in nude mice with tumors. As shown in the figure, the fluorescence intensity at the tumor site was the strongest 2 h after tail vein administration. This indicates that FITC-labeled GPDD accumulates the most at the tumor site at this time. At 8 h, the fluorescence intensity of other tissues in the body became weak, while there was strong fluorescence at the tumor site, indicating that GE11-PEG-hyd-DOX/DOX can accumulate at the tumor site instead of other tissues and organs, and its metabolism in the body is relatively slow. It has a good sustained release effect and certain tumor targeting.

### 3.8. Experimental Results of Drug Distribution In Vivo

According to the experimental results shown in Figure 9B,C, DOX was rapidly distributed in the heart, liver, spleen, lung, and kidney after tail vein administration in nude mice, with little difference in drug distribution in the spleen and lung. The drug concentration at the tumor site in the free DOX administration group was significantly lower than that in the GPDD group, indicating that GPDD can enhance the tumor targeting of drugs. By comparing DOX concentrations in various organs of the two drug administration groups, it can be seen that the free DOX administration group can enrich DOX in the heart to a certain extent, while GPDD can reduce the concentration of DOX in the heart and reduce the cardiotoxicity of DOX to a certain extent, which is consistent with the results of in vivo targeting experiments.

### 3.9. In Vivo Antitumor Efficiency

Table 10 shows the tumor suppression of tumor-bearing nude mice in different administration groups after 3 weeks of continuous tail vein administration, including weight changes in nude mice before and after administration, tumor weights of nude mice after administration, tumor suppression rate (TGI), and spleen index of tumor-bearing nude mice in each group. Figure 9D shows the tumor growth curves of each group. The tumors in the control group grew most rapidly, and their volume reached 1168.83 mm^3^ at the end of treatment. The volume of the free DOX group was 557.48 mm^3^. The volume of the PDD group was 448.43 mm^3^. In the GPDD drug-loaded micelle group, the tumor growth was the slowest, and the tumor volume was only 162.03 mm^3^, which was significantly different from that in the control group (*p* < 0.01).

As illustrated in Figure 9E, body weight trajectories across treatment groups revealed a marked decline in the free DOX cohort throughout the dosing period, a reduction that reached statistical significance relative to the control group (*p* < 0.01). Concurrently, animals in this group exhibited diminished food and water consumption, progressive cachexia, and reduced locomotor activity. These adverse phenotypes are likely attributable to the systemic toxicity inherent to unencapsulated DOX. In contrast, no statistically significant deviations in body weight were observed between the control group and the two micelle-formulated groups, suggesting that encapsulation within drug-loaded micelles effectively mitigates, at least partially, the systemic toxic effects associated with free DOX administration.

As illustrated in Figure 9F, an examination of excised tumors from nude mice across all treatment cohorts revealed that the GPDD-loaded micelle group exhibited markedly reduced tumor volumes compared to the other three groups. Quantitative analysis of post-treatment tumor masses, depicted in Figure 9G, further confirmed that the GPDD formulation achieved a significantly lower tumor burden relative to the control group, which recorded a mean weight of 1.076 ± 0.12 g. Specifically, the free DOX cohort demonstrated a tumor mass of 0.75 ± 0.05 g, corresponding to an inhibition rate of 29.93%, while the PDD group showed a reduced weight of 0.65 ± 0.06 g with a 39.78% inhibition rate. Notably, the GPDD group attained the lowest tumor weight at 0.27 ± 0.07 g, yielding a substantial inhibition rate of 75.10%. Collectively, these data underscore the potent antitumor efficacy of GPDD-loaded micelles in A549 xenograft nude mouse models.

The results of the spleen index (Figure 9H) of each group showed that SI in the free DOX group (*p* < 0.01) and PDD group (*p* < 0.05) was significantly lower than that in the control group. There was no significant difference between the GPDD drug-loaded micelle group and the control group (Figure 9I).

### 3.10. Pathological Observations of Tumor and Major Organs

#### 3.10.1. HE Staining Experiment

**Figure 10:**
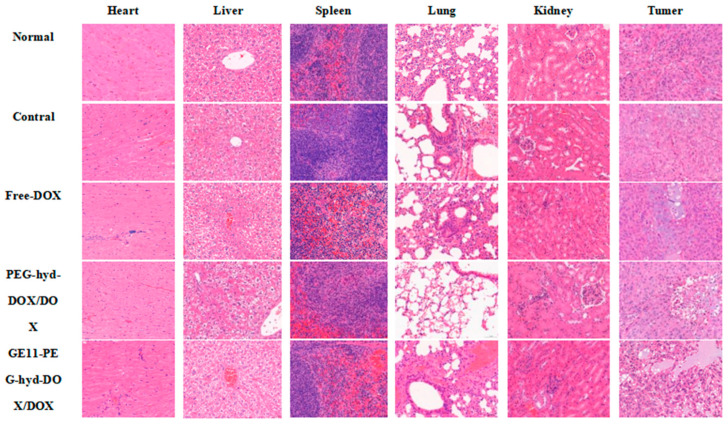
HE staining results of various tissues in nude mice (400×). Bar, 50 µm.

Histopathological changes were observed (Figure 10) under the Moticam3000 photomicrographic imaging system, and the film was taken 400 times under microscope. Histopathological changes in different drug administration groups were analyzed by histopathological sections of tumorous nude mice. The number of spleen lymphocytes decreased and mild inflammation occurred in the free DOX group, while the heart, liver, spleen, lung and kidney of nude mice in the control group, normal group, PDD group and GPDD group all maintained their original tissue morphology, and no obvious histopathological lesions appeared. Tumor tissues of the free DOX group, PDD group and GPDD group showed different degrees of cell degeneration and necrosis, indicating that the GPDD group had stronger toxicity to tumor cells and minimal systemic toxicity to nude mice.

#### 3.10.2. TUNEL Staining Experiment

**Figure 11:**
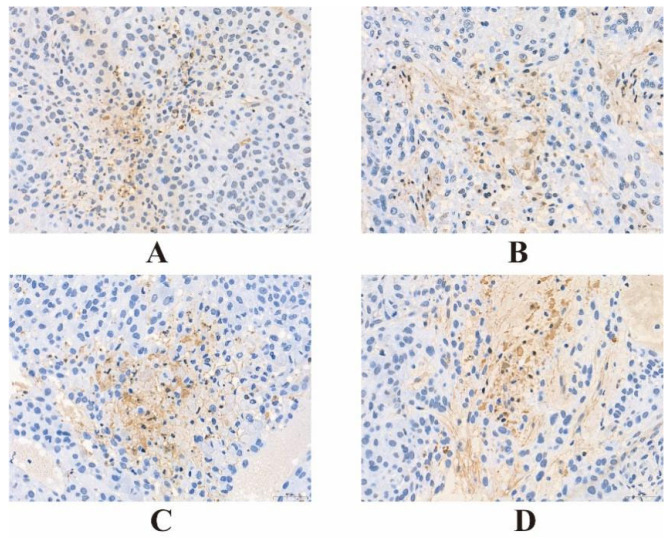
TUNEL staining results of tumor tissue. Bar, 50 µm. (**A**) Control, (**B**) Free DOX, (**C**) PEG-hyd-DOX/DOX, (**D**) GE11-PEG-hyd-DOX/DOX.

The Pannoramic 250 digital slicing scanner was used to collect images from slices. All tissues of each slice were observed at low magnification first, and then three representative areas were selected according to their expression to collect 400-fold images, as shown in Figure 11. The Image-pro Plus 6.0 Image analysis system was used to measure the internal area of the collected images and calculate the apoptosis index. The results are shown in Table 11. The results showed that the three drug administration groups had significant differences regarding the apoptosis of tumor cells in nude mice compared with the control group (*p* < 0.01). Compared with the free DOX group, the AI value of the PDD group and GPDD group was significantly increased (*p* < 0.01), indicating that all three dosage forms could produce apoptosis of tumor cells, but GPDD drug-loaded micelles promoted apoptosis more strongly.

## 4. Conclusions

This study successfully constructed a GE11-modified pH-sensitive polymeric micelle targeted drug delivery system for the targeted treatment of non-small-cell lung cancer (NSCLC). The prepared GPDD and PDD exhibited high drug-loading capacity and excellent internal and external stability. In vitro, PDD and GPDD significantly increased the uptake of DOX by A549 cells, leading to tumor cell death. According to in vivo imaging results, due to the presence of GE11, GPDD easily accumulated at the tumor sites in tumor-bearing animals, demonstrating good tumor targeting. Ultimately, in a tumor-bearing mouse model, GPDD showed a relatively high tumor growth inhibition effect and reduced systemic toxicity. However, due to the autofluorescence issue of DOX, the critical micelle concentration of the intact formulation was not determined, which presents certain limitations. This study proposes an efficient and low-toxicity pH-sensitive polymeric micelle targeted drug delivery system, in which the EGFR-targeted GE11 delivers the drug to the tumor site, decomposes in the tumor microenvironment, and releases the drug, effectively destroying tumor cells, indicating that GPDD has great potential in targeted drug delivery.

## Figures and Tables

**Table 1:** Result of the coupling rate of GE11 (*n* = 3).

No.	1	2	3	Mean ± SD	RSD (%)
Coupling rate (%)	87.23	88.06	88.71	88.00 ± 0.61	0.70

**Table 2:** The EE and DL of GPDD and G of GPD (*n* = 3).

No.	1	2	3	Mean ± SD	RSD (%)
EE (%)	78.15	80.63	80.38	79.72 ± 1.11	1.39
DL (%)	3.84	4.06	3.95	3.95 ± 0.09	2.28
G (%)	17.98	18.30	18.76	18.35 ± 0.32	1.74

**Table 3:** The result of the stability of micelles (*n* = 3).

Time (h)	0	4	8	12	24	RSD (%)
Mean ± SD (μg·mL^−1^)	4.98 ± 0.07	4.96 ± 0.08	4.95 ± 0.04	4.93 ± 0.09	4.89 ± 0.04	0.61

**Table 4:** Results of hemolytic test.

Group	1	2	3	4	5	6	7
2%Red blood cell suspension	2.5	2.5	2.5	2.5	2.5	2.5	2.5
Saline	2.5	2.4	2.3	2.2	2.1	2.0	0
Distilled water	0	0	0	0	0	0	2.5
GPDD	0	0.1	0.2	0.3	0.4	0.5	0
Hemolysis result	-	-	-	-	-	-	++

**Table 5:** Fitting equation of the accumulative release percentage of DOX (pH = 7.4).

Model	Drugs	Equation of Fit	R^2^
Zero-order kinetic equations were fitted	Free DOX	M_t_ = 4.3112k + 55.7550	0.4813
	GPDD	M_t_ = 0.3338 + 28.3725	0.5273
First-order kinetic equation was fitted	Free DOX	M_t_ = 90.8056 (1 − e^−1.5803t^)	0.9248
	GPDD	M_t_ = 40.9365 (1 − e^−1.0450t^)	0.6719
Higuchi Equation fitting	Free DOX	M_t_ = 19.4113x^1/2^ − 39.8081	0.6651
	GPDD	M_t_ = 3.4760x^1/2^ + 22.9049	0.7735
Weibull Equation fitting	Free DOX	M_t_ = 116.5859 (1 − e^(−(0.9356(x−0.2471))0.2493)^)	0.9985
	GPDD	M_t_ = 47.0015 (1 − e^(−(0.3529(x+0.4184))0.4809)^)	0.9897
Ritger–Peppas Equation fitting	Free DOX	M_t_ = 61.0270x^0.2157^	0.7824
	GPDD	M_t_ = 25.5550x^0.1574^	0.9383

**Table 6:** Fitting equation of the accumulative release percentage of DOX (pH = 6.8).

Model	Drugs	Equation of Fit	R^2^
Zero-order kinetic equations were fitted	Free DOX	M_t_ = 4.3073k + 55.5173	0.4751
	GPDD	M_t_ = 0.5128k + 36.7386	0.5042
First-order kinetic equation was fitted	Free DOX	M_t_ = 90.2208 (1 − e^−1.6126t^)	0.9037
	GPDD	M_t_ = 57.5219 (1 − e^−0.7585t^)	0.8434
Higuchi Equation fitting	Free DOX	M_t_ = 19.3567x^1/2^ + 39.6482	0.6541
	GPDD	M_t_ = 5.35777x^1/2^ + 28.2791	0.7478
Weibull Equation fitting	Free DOX	M_t_ = 155.3473 (1 − e^(−(0.0823(x−0.2494))0.1844)^)	0.9985
	GPDD	M_t_ = 66.1892 (1 − e^(−(0.3778(x−0.0909))0.4071)^)	0.9955
Ritger–Peppas Equation fitting	Free DOX	M_t_ = 60.8079x^0.2158^	0.7685
	GPDD	M_t_ = 32.5527x^0.1805^	0.9167

**Table 7:** Fitting equation of the accumulative release percentage of DOX (pH = 5.0).

Model	Drugs	Equation of Fit	R^2^
Zero-order kinetic equations were fitted	Free DOX	M_t_ = 4.2500k + 56.0634	0.4465
	GPDD	M_t_ = 0.7392k + 41.9592	0.6353
First-order kinetic equation was fitted	Free DOX	M_t_ = 0.8795 (1 − e^−1.6907t^)	0.8795
	GPDD	M_t_ = 69.7526 (1 − e^−0.7043t^)	0.7333
Higuchi Equation fitting	Free DOX	M_t_ = 19.0927x^1/2^ + 40.4160	0.6173
	GPDD	M_t_ = 7.4117x^1/2^ + 30.7699	0.8462
Weibull Equation fitting	Free DOX	M_t_ = 353.5306 (1 − e^(−(1.6699(x−0.2500))0.1322)^)	0.9951
	GPDD	M_t_ = 117.6683 (1 − e^(−(0.0357(x−0.1932))0.2706)^)	0.9787
Ritger–Peppas Equation fitting	Free DOX	M_t_ = 61.3002x^0.2121^	0.7310
	GPDD	M_t_ = 36.9821x^0.2049^	0.9546

**Table 8:** The immunohistochemical IOD values for tumor tissue (*n* = 3).

Group	p62	Bcl-2	Bax
Control	0.84 ± 0.04	0.94 ± 0.05	0.20 ± 0.01
Free DOX	0.45 ± 0.02 **	0.73 ± 0.04 **	0.52 ± 0.02 **
PEG-hyd-DOX/DOX	0.44 ± 0.01 **	0.62 ± 0.02 *^#^	0.74 ± 0.04 **^#^
GE11-PEG-hyd-Dox/Dox	0.37 ± 0.02 **^##^	0.40 ± 0.01 **^##^	1.09 ± 0.03 **^##^

* *p* < 0.05, ** *p* < 0.01, compared with control group; ^#^ *p* < 0.05, ^##^ *p* < 0.01, compared with the free DOX group.

**Table 9:** IC_50_ of free DOX, PDD and GPDD (*n* = 6).

T (h)	IC_50_ (μg·mL^−1^)		
	Free-DOX	PDD	GPDD
24	17.712 ± 0.871	25.078 ± 1.618 **	23.884 ± 0.475 **
48	6.330 ± 0.539	11.352 ± 0.445 **	6.570 ± 0.295 ^##^
72	4.886 ± 0.474	7.632 ± 0.897 **	3.708 ± 0.495 *^##^

* *p* < 0.05, ** *p* < 0.01, PDD, GPDD compared with DOX. ^##^ *p* < 0.01, GPDD compared with PDD.

**Table 10:** Tumor inhibition rate of different group after administration (*n* = 5).

Group	Body Weight (g)		Tumor Weight (g)	TGI (%)	SI (mg/g)
	Before	After			
Control	19.46 ± 1.27	22.35 ± 1.58	1.08 ± 0.12	-	8.51 ± 0.63
Free DOX	19.75 ± 0.99	16.68 ± 0.57	0.75 ± 0.05	29.93	4.32 ± 0.35
PEG-hyd-DOX/DOX	19.88 ± 0.72	21.64 ± 1.18	0.65 ± 0.06	39.78	6.20 ± 0.44
GE11-PEG-hyd-DOX/DOX	19.43 ± 0.98	21.80 ± 1.15	0.27 ± 0.07	75.10	7.80 ± 0.53

**Table 11:** Apoptosis index of nude mice tumor cells (*n* = 5).

Group	AI (%)
Control	9.13 ± 2.79
Free DOX	19.92 ± 5.06 **
PEG-hyd-DOX/DOX	21.65 ± 3.74 **
GE11-PEG-hyd-DOX/DOX	34.41 ± 4.56 **^##^

** *p* < 0.01, free DOX, PDD, GPDD separately compared with control; ^##^ *p* < 0.01, PDD, GPDD separately compared with free DOX.

## Data Availability

The original contributions presented in this study are included in the article. Further inquiries can be directed to the corresponding author.
